# Comparison of zeta potential and physiological intracytoplasmic sperm injection in obtaining sperms with a lower DNA fragmentation index: A cross-sectional study

**DOI:** 10.18502/ijrm.v20i5.11050

**Published:** 2022-06-08

**Authors:** Serajoddin Vahidi, Nima Narimani, Laleh Dehghan Marvast, Esmat Mangoli, Ali Nabi, Mohammad Sadeghi

**Affiliations:** ^1^Andrology Research Center, Yazd Reproductive Sciences Institute, Shahid Sadoughi University of Medical Sciences, Yazd, Iran.; ^2^Department of Urology, Hasheminejad Kidney Center (HKC), Iran University of Medical Sciences (IUMS), Tehran, Iran.

**Keywords:** PICSI, ZETA potential, Hyaluronic acid, DNA integrity.

## Abstract

**Background:**

The sperm DNA fragmentation index (DFI) is one of the men's reproductive health criteria that affects assisted reproductive technique outcomes. Efforts in obtaining high-quality mature sperms seem to be necessary. Advanced sperm selection techniques (including physiological intracytoplasmic sperm injection [PICSI], zeta potential, microfluidic, etc.) have gained popularity in this regard.

**Objective:**

The study aimed to compare the efficacy of zeta potential and PICSI sperm selection in obtaining sperms with better DNA integrity.

**Materials and Methods:**

In this cross-sectional study, 48 couples were enrolled where the male partner had increased sperm DFI in his ejaculated sample and the female was in normal reproductive health. For each male partner, the semen sample was processed with zeta potential and PICSI techniques, then the sperm DFI of neat semen was compared to zeta and PICSI samples by the sperm chromatin dispersion test.

**Results:**

Data showed that both the zeta potential and PICSI technique decreased sperm DFI in comparison with the neat semen sample (p < 0.001 for both). In addition, there was a statistically significant difference in sperm DFI between the PICSI and zeta potential samples (p < 0.01).

**Conclusion:**

The current study showed that both zeta potential and PICSI could result in sperm with a lower DFI. However, PICSI seems to be superior to zeta potential in this regard.

## 1. Introduction

According to the World Health Organization definition, about 15% of couples face infertility and its related problems (1). Male fertility potential is traditionally assessed with a semen analysis, which is considered a cornerstone in the andrology clinic. However, about 15% of infertile men may have normal semen analysis (2). Assessment of sperm DNA damage seems to be necessary for such patients. Previous studies have suggested that an increased sperm DNA fragmentation index (DFI) may lead to lower natural and intrauterine insemination pregnancy rates as well as compromised assisted reproduction technique (ART) outcomes (3, 4). There are reports about the temporal decline in semen quality and also increased use of ART techniques, worldwide (1, 5). Obtaining functionally competent sperms with the highest DNA integrity seems to be necessary to increase the ART success rate. Management of increased sperm DFI may include treatment of underlying causes (such as varicocelectomy, male accessory gland infections, lifestyle change, etc.), practical use of antioxidants, short abstinence, sperm selection techniques, and the use of retrieved testicular sperms (6, 7).

Contrary to routine intracytoplasmic sperm injection (ICSI), in which sperms are selected based on their normal morphology and motility, advanced sperm selection techniques were developed to determine functionally mature sperms with lower DFI. These techniques are diverse and include physiological intracytoplasmic sperm injection (PICSI), magnetic-activated cell sorting (MACS), zeta potential, motile sperm organelle morphology examination and intracytoplasmic morphologically selected sperm injection (MSOME & IMSI), etc. (8).

Naturally, the human oocyte is surrounded by hyaluronic acid (HA). It is proposed that only mature sperm may gain the ability to bind to this natural barrier. These sperms may have a lower aneuploidy rate and better DNA integrity (9). The PICSI technique is based on in vitro sperm selection using a plastic culture dish containing HA dots (10). This technique reduces sperm motility by encouraging sperm attachment to HA dots (in contrast to routine ICSI in which the aforementioned goal is achieved by using polyvinylpyrrolidone (PVP) (9). Given that PVP may have a toxic effect on spermatozoa (11), PICSI may result in obtaining sperm with better DNA integrity.

Zeta potential is based on the fact that mature spermatozoa have a negative membrane electrical charge (-16 mV to -22 mV). The zeta potential method may lead to obtaining sperm with better DNA integrity as well as embryo development (12, 13). So far, according to a PubMed search, there has only been a single study which has compared the efficacy of PICSI and zeta potential methods on sperm function tests (14).

In the current study, we therefore aimed to compare the efficacy of PICSI sperm selection over zeta potential in obtaining sperms with better DNA integrity and chromatin compaction.

## 2. Materials and Methods

70 men who referred to Research and Clinical Center for Infertility (Yazd, Iran) between November 2019 and March 2021 were enrolled in this cross-sectional study. The inclusion criteria were infertile couples with a history of recurrent pregnancy loss (more than 2 miscarriages in the 1 ${}^{\text{st}}$ trimester) and ART failures ( ≥ 2 in vitro fertilization or ICSI failures) where the male partner had increased sperm DFI in the ejaculated samples (DFI > 30% assessed by sperm chromatin dispersion test [SCD]) and where the female partner presented a normal workup. Other criteria were: male partner younger than 45 yr, progressive sperm count more than 10 million/ml (10), and female partner < 35 yr old.

Exclusion criteria were moderate to severe oligospermia (sperm count < 5 million/ml), progressive motility < 5%, modifiable risk factors for increased sperm DFI such as smoking, previous history of unsafe intercourse, varicocele, undescended testis, and known hormonal derangement. Finally, 22 cases were excluded, and those remaining were enrolled in the current study. The same urologist performed history taking and physical examination.

### Sample collection 

Semen samples were collected after 3 days of abstinence from intercourse by masturbation. Each semen sample was divided into 3 parts: the 1 ${}^{\text{st}}$ part (group 1) included the unprocessed semen sample considered as the control group, part 2 and 3 (group 2 and 3) were processed by the density gradient method and then sperm selection was made with PICSI and zeta potential, respectively.

Semen analysis was performed based on World Health Organization guidelines (15). Sperm morphology was analyzed based on strict criteria. Motility was reported as a percentage of progressive, non-progressive, and immotile sperms. Also, sperm DNA integrity was analyzed by SCD.

### SCD 

The SCD test was used to assess sperm DNA fragmentation. This test was carried out according to the sperm DNA fragmentation assay kit protocol (Ideh varzan farda, Tehran, Iran). In brief, 50 µl of sperm suspension was mixed with 100 µl low melting agarose (Roche, Germany). 30 µl of the prepared mixture was placed on precoated slides. They were put at 4 C for 4 min. Then, solution A was used to immerse slides for 7 min at room temperature (RT) in the dark. The slides were placed into solution B for 15 min at RT. After that, the slides were washed for 5 min in sterile water. 70%, 90%, and 100% ethanol were used (2 min each) to dehydrate the samples, and then they were left to dry at RT. For staining, solutions C, D and E were used for 2, 3, and 4 min, respectively. Eventually, the slides were evaluated by a light microscope (Olympus, Japan). The DNA fragmentation rate was calculated based on different halo patterns. Based on a previous study, large or medium-sized halos that appeared around sperm cells showed sperm without DNA fragmentation, while small or no halos presented sperm with DNA fragmentation (16).

### Zeta potential

The technique was performed similar to the protocol previously described else where (17). After being processed by density gradient centrifugation, the sample pellets were washed with Ham's F-10 (without albumin). The pellets were diluted with 4 ml of Ham's-F 10 and resuspended in the Falcon plastic tubes (5 ml). The technique is based on exposure of the sperms to the positive electrical charge. For this goal, after being placed in the latex glove, the Falcon tube was twisted (or totated) 2 or 3 turns and rapidly removed from the glove. The tube was kept in the RT for 1 min (to allow the sperms with high quality to adhere to the charged tube wall). The medium containing non-adhering sperms was dispended from the tube. In order to neutralizing the positive charge and retrieval of adhered sperms, the tube wall was washed with 4 ml of Ham's F-10 plus albumin (5 ml). The tube was centrifugated at 300 g for 5 min. The pellet was re-suspended in Ham's F-10 plus albumin (1 ml) for use in ICSI.

### PICSI sperm selection

To select the morphologically `best' spermatozoa, PICSI dishes were used. PICSI dishes (Biocoat, Inc., Horsham, PA, USA) were prepared by hydrating the hyaluronan microdots with 10 μl of Ham's-F10 medium supplemented with 5 mg albumin and covered with 3-4 ml light mineral oil (Irvine Scientific, Santa Ana, CA, USA). We placed the final sperm suspension into droplets of Ham's-F10 medium, and selected spermatozoa that bounded to HA microdots and separated them with an injecting pipette (ICSI Micropipette; ORIGIO, Charlottesville, VA, USA) and subsequently loaded them into 5 µl low-melting-point agarose gels (which were deposited onto the pre-coated slide) for determining the sperm DNA fragmentation (18, 19).

### Ethical considerations

The Institutional Review Board and Ethics Committee of Shahid Sadoughi University of Medical Science, Yazd, Iran approved the study protocol (Code: IR.SSU.MEDICINE.REC.1398.176). Written consent was obtained from all the participants after being fully informed.

### Statistical analysis

Data are shown as mean ± standard deviation, median (interquartile range), range for quantitative variables and frequency (percentage) for qualitative ones. The Kolmogorov-Smirnov test was used to assess the normality of data distribution. According to the normality test results, the Friedman test was used to compare the mean outcome quantities between zeta potential, a neat semen sample, and PICSI DFI. Multiple comparison was done by the Mann-Whitney U test and the significance level was adjusted using the Bonferroni correction. The Pearson and Spearman tests were also used to assess the correlation between the qualitative factors. The statistical analyses were performed using the Statistical Package for the Social Sciences software (SPSS) version 24.0 (stocktickerIBM, Chicago, Illinois, USA). A p-value < 0.05 was considered statistically significant.

## 3. Results

A total of 48 men were enrolled in the current study. The mean age and body mass index of participants were 33.93 ± 7.20 yr and 23.33 ± 2.81 kg/m^2^, respectively. The results of the semen parameters are depicted in table I, and the sperm DFI for the neat semen samples, PICSI and zeta potential, and also the comparison between them, are presented in table II. The data showed that sperm DFI was significantly reduced with sperm selection techniques (p < 0.001).

Also, table III shows the correlation between neat semen DFI with male age and other semen parameters. There was a significant positive correlation between age and neat semen DFI (p = 0.007) and a significant negative correlation between normal morphology and neat semen DFI (p = 0.045).

**Table 1:** Characteristic of the neat semen samples


**Semen parameters**	**Mean ± SD**	**Range (min-max)**	**Median (IQR)**
**Sperm counts (million/ml)**	68.27 ± 36.50	10.0-150.0	67.5 (39.0, 97.5)
**Progressive motility (%)**	35.35 ± 13.15	10.0-60.0	32.5 (26.5, 46.0)
**Non-progressive motility (%)**	11.60 ± 4.56	5.0-25.0	10.0 (8.0, 15.0)
**Immotile sperms (%)**	53.04 ± 12.02	30.0-80.0	54.0 (43.5, 60.0)
**Normal morphology (%)**	3.23 ± 1.61	1.0-7.0	3.0 (2.0, 4.0)
IQR: Interquartile range			

**Table 2:** Comparison of sperm DNA fragmentation between neat semen, zeta potential, and PICSI methods


**Variables **	**Mean ± SD**	**Median (IQR)**	**Range (min-max)**	**P-value**
**Neat semen sample DFI**	33.00 ± 4.83 ${}^{ab}$	32.5 (29.0, 36.0)	25.0-43.0	
**Zeta potential DFI**	28.04 ± 5.64 ${}^{ac}$	28.0 (24.0, 30.50)	18.0-42.0	
**PICSI DFI**	24.15 ± 4.82 ${}^{bc}$	24.0 (20.5, 28.0)	15.0-35.0	< 0.001
With post hoc comparison p-value based on Bonferroni correction for multiple tests as follows: ${}^{a}$ P-value < 0.001, ${}^{b}$ P-value < 0.001, ${}^{c}$ P-value < 0.01. DFI: DNA fragmentation index, PICSI: Physiological intracytoplasmic sperm injection, IQR: Interquartile range				

**Table 3:** Correlation between age and semen parameters with neat semen DFI


	**Neat semen sample DFI**	
	**Correlation**	**P-value**
	**Male age **	0.385	0.01 ${}^{*}$
**Sperm count **	-0.268	0.07
**Progressive motility**	-0.192	0.19
**Non-progressive motility **	0.069	0.64
**Immotile sperms**	0.184	0.21
**Normal morphology**	-0.290	< 0.05 ${}^{*}$
The Pearson and Spearman tests were used. *P-value < 0.05 was significant. DFI: DNA fragmentation index		

## 4. Discussion

The current study showed that sperm selection techniques might be beneficial in obtaining sperm with better DNA integrity. As stated in the results section, both the zeta potential and PICSI techniques could significantly improve sperm DNA integrity in comparison with neat semen samples. The findings also showed that sperm selection by HA can result in sperms with a lower DFI compared to those from zeta potential. To the best of our knowledge, there is only a single study in PubMed comparing PICSI and zeta potential in terms of obtaining sperm with better DNA integrity (14).

Our results are not in agreement with another similar study that stated the superiority of zeta potential over hyaluronic sperm selection in terms of sperm DNA integrity. This difference may be partly due to differences in HA-selection tools and the role of an embryologist in sperm selection. In our study, by using a standard PICSI dish (PICSI dish; Biocoat, Inc., Horsham, PA, USA), seemingly suitable sperm were selected for injection by an embryologist. They were collected in a tiny dot of sperm wash (on an agarose-coated slide), and their DNA integrity was assessed with an SCD test. Meanwhile, the aforementioned study evaluated the DNA integrity of unselected HA-bound sperms on a handmade slide coated with HA. We believe that the role of an embryologist in sperm selection and the use of a standard PICSI dish may have led to the aforementioned difference in findings. However, this difference may also be due to our small sample size or the small number of selected sperms. Another recent study compared PICSI with MACS techniques in selecting spermatozoa with lower DNA fragmentation. This study showed both of these techniques were effective in sperm selection, but intimated that MACS was preferential in cases with younger women and PICSI was more effective in the cases with older ones (19).

Since advanced sperm selection methods were developed comparatively recently, assessment of their efficacy seems to be necessary. It has been proposed that PVP, used in ICSI procedures, may have a toxic effect on spermatozoa (11, 20). PVP may result in sperm membrane injury and embryonal maldevelopment (20). It has been shown that this negative effect may be dose-dependent (21). Whereas PVP is widely used in zeta potential to reduce sperm motility, such a goal is achieved by a more physiologic substitute (HA) in the PICSI technique (9).

The hyaluronic-based sperm selection technique was developed to obtain mature sperm. As mentioned before, some authors suggest that only mature functional sperms might gain the ability to bind to the HA receptors. These sperm usually show a high degree of nuclear and cytoplasmic maturity (22). However, the effect of HA-bound sperms on ART outcome remains the subject of debate. A prospective study on 232 ICSI cycles randomized to PVP-ICSI (107 cycles) and HA-selected sperms (125 cycles), showed better embryo development in the latter group (23). However, British scientists performed a well-designed randomized trial on 2772 couples and their study indicated no statistically significant difference between PICSI and conventional ICSI in terms of clinical pregnancy and live birth rate (24).

Recently, another study stated that PICSI might significantly increase the fertilization rate and embryo transfer in couples with previous ART failures compared to routine ICSI. The authors concluded that this technique should be performed with infertile couples with a history of unsuccessful IVF cycles (25). The current study showed statistically significant differences between PICSI and zeta potential in obtaining sperms with better DNA integrity. However, the clinical significance of this finding in terms of ART outcome is yet to be defined and should be evaluated in upcoming studies.

Sperm chromatin is well-organized and has high nuclear condensation, and any alteration of it during spermatogenesis could have harmful effects on sperm functions (26). As shown in table III, there is a positive correlation between male age and neat sperm DFI. This means that advanced paternal age may negatively affect sperm DNA integrity (increased sperm DFI). This result is in line with recent studies which showed that increased male age might result in decreased sperm DNA quality (27, 28). This effect may be due to increased reactive oxygen species production, defective sperm chromatin compaction, disordered apoptosis, and changes in telomer length (29). Also, it is widely accepted that sperm DNA fragmentation may influence the semen quality (26, 30). Among the sperm characteristics, sperm morphology may have a crucial role in the diagnosis of male fertility potential. It means that abnormally shaped spermatozoa may have increased rate of chromosomal aneuploidy, a higher DFI, and an increased chance of mitochondrial dysfunction (31). In this regard, this study's data showed a significant negative correlation between normal morphology spermatozoa and sperm DFI. This result aligns with earlier studies suggesting that normal morphology sperm selected with motile sperm organelle morphology examination and intracytoplasmic morphologically selected sperm injection had significantly lower DFI than those from ICSI (32).

There are other limitations in the current study. Since the number of embryologist-selected sperms were limited, only sperm DNA integrity could be assessed, and the status of sperm chromatin compaction remained unclear. Further attempts to select a larger number of sperms may result in increased sperm DFI due to prolongation of the process.

Finally, given the small number of participants included in the current study, the results should be evaluated with larger samples in future studies.

## 5. Conclusion

The current study showed that advanced sperm selection techniques may lead to obtaining sperms with better DNA integrity. In this regard. PICSI may result in sperms with higher quality in comparison with zeta potential. However, further studies are needed to determine the clinical significance of this finding.

##  Conflict of Interest

The authors declare that there is no conflict of interest.

## References

[B1] Vahidi S, Moein MR, Yazdinejad F, Ghasemi-Esmailabad S, Narimani N (2020). Iranian temporal changes in semen quality during the past 22 years: A report from an infertility center. Int J Reprod Biomed.

[B2] Agarwal A, Allamaneni SSR (2005). Sperm DNA damage assessment: A test whose time has come. Fertil Steril.

[B3] Deng C, Li T, Xie Y, Guo Y, Yang QY, Liang X, et al (2019). Sperm DNA fragmentation index influences assisted reproductive technology outcome: A systematic review and meta-analysis combined with a retrospective cohort study. Andrologia.

[B4] Zini A

[B5] Ionov M, Gontarek W, Bryszewska M (2020). Zeta potential technique for analyzing semen quality. MethodsX.

[B6] Agarwal A, Majzoub A, Baskaran S, Panner Selvam MK, Cho ChL, Henkel R, et al (2020). Sperm DNA fragmentation: A new guideline for clinicians. World J Mens Health.

[B7] Vahidi S, Narimani N, Ghanizadeh T, Yazdinejad F, Emami M, Mehravaran K, et al

[B8] Pinto S, Carrageta DF, Alves MG, Rocha A, Agarwal A, Barros A, et al (2021). Sperm selection strategies and their impact on assisted reproductive technology outcomes. Andrologia.

[B9] Parmegiani L, Cognigni GE, Ciampaglia W, Pocognoli P, Marchi F, Filicori M (2010). Efficiency of hyaluronic acid (HA) sperm selection. J Assist Reprod Genet.

[B10] Erberelli RF, Salgado RM, Pereira DHM, Wolff Ph (2017). Hyaluronan-binding system for sperm selection enhances pregnancy rates in ICSI cycles associated with male factor infertility. JBRA Assist Reprod.

[B11] Jean M, Mirallie S, Boudineau M, Tatin C, Barriere P (2001). Intracytoplasmic sperm injection with polyvinylpyrrolidone: A potential risk. Fertil Steril.

[B12] Duarte C, Nunez V, Wong Y, Vivar C, Benites E, Rodriguez U, et al (2017). Impact of the Z potential technique on reducing the sperm DNA fragmentation index, fertilization rate and embryo development. JBRA Assist Reprod.

[B13] Ghorbani-Sini R, Izadi T, Tavalaee M, Azadi L, Hajian M, Rahimi Zamani M, et al (2020). Comparison of sperm telomere length between two sperm selection procedures: Density gradient centrifugation and zeta potential. Int J Fertil Steril.

[B14] Razavi SH, Nasr‐Esfahani MH, Deemeh MR, Shayesteh M, Tavalaee M (2010). Evaluation of zeta and HA‐binding methods for selection of spermatozoa with normal morphology, protamine content and DNA integrity. Andrologia.

[B15] Cooper TG, Noonan E, von Eckardstein S, Auger J, Baker HWG, Behre HM, et al (2009). World health organization reference values for human semen characteristics. Hum Reprod Update.

[B16] Nabi A, Khalili MA, Fesahat F, Talebi A, Ghasemi-Esmailabad S (2017). Pentoxifylline increase sperm motility in devitrified spermatozoa from asthenozoospermic patient without damage chromatin and DNA integrity. Cryobiology.

[B17] Nasr Esfahani MH, Deemeh MR, Tavalaee M, Sekhavati MH, Gourabi H (2016). Zeta sperm selection improves pregnancy rate and alters sex ratio in male factor infertility patients: A double-blind, randomized clinical trial. Int J Fertil Steril.

[B18] Mokanszki A, Tothne EV, Bodnar B, Tandor Z, Molnar Z, Jakab A, et al (2014). Is sperm hyaluronic acid binding ability predictive for clinical success of intracytoplasmic sperm injection: PICSI vs. ICSI? Syst Biol Reprod Med.

[B19] Hasanen E, Elqusi K, ElTanbouly S, Hussin AE, AlKhadr H, Zaki H, et al (2020). PICSI vs. MACS for abnormal sperm DNA fragmentation ICSI cases: A prospective randomized trial J Assist Reprod Genet.

[B20] Kato Y, Nagao Y (2012). Effect of polyvinylpyrrolidone on sperm function and early embryonic development following intracytoplasmic sperm injection in human assisted reproduction. Reprod Med Biol.

[B21] Ding D, Wang Q, Li X, Chen B, Zou W, Ji D, et al (2020). Effects of different polyvinylpyrrolidone concentrations on intracytoplasmic sperm injection. Zygote.

[B22] Huszar G, Jakab A, Sakkas D, Ozenci CC, Cayli S, Delpiano E, et al (2007). Fertility testing and ICSI sperm selection by hyaluronic acid binding: Clinical and genetic aspects. Reprod Biomed Online.

[B23] Parmegiani L, Cognigni GE, Bernardi S, Troilo E, Ciampaglia W, Filicori M (2010). "Physiologic ICSI": Hyaluronic acid (HA) favors selection of spermatozoa without DNA fragmentation and with normal nucleus, resulting in improvement of embryo quality. Fertil Steril.

[B24] Miller D, Pavitt S, Sharma V, Forbes G, Hooper R, Bhattacharya S, et al (2019). Physiological, hyaluronan-selected intracytoplasmic sperm injection for infertility treatment (HABSelect): A parallel, two-group, randomised trial. Lancet.

[B25] Novoselsky Persky M, Hershko-Klement A, Solnica A, Bdolah Y, Hurwitz A, Ketzin El Gilad M, et al (2021). Conventional ICSI vs. physiological selection of spermatozoa for ICSI (picsi) in sibling oocytes Andrology.

[B26] Sun TCh, Zhang Y, Li HT, Liu XM, Yi DX, Tian L, et al (2018). Sperm DNA fragmentation index, as measured by sperm chromatin dispersion, might not predict assisted reproductive outcome. Taiwan J Obstet Gynecol.

[B27] Rosiak-Gill A, Gill K, Jakubik J, Fraczek M, Patorski L, Gaczarzewicz D, et al (2019). Age-related changes in human sperm DNA integrity. Aging (Albany NY).

[B28] Kaarouch I, Bouamoud N, Madkour A, Louanjli N, Saadani B, Assou S, et al (2018). Paternal age: Negative impact on sperm genome decays and IVF outcomes after 40 years. Mol Reprod Dev.

[B29] Petersen CG, Mauri AL, Vagnini LD, Renzi A, Petersen B, Mattila M, et al (2018). The effects of male age on sperm DNA damage: An evaluation of 2,178 semen samples. JBRA Assist Reprod.

[B30] Mangoli E, Khalili MA, Talebi AR, Ghasemi-Esmailabad S, Hosseini A

[B31] Ferrigno A, Ruvolo G, Capra G, Serra N, Bosco L (2021). Correlation between the DNA fragmentation index (DFI) and sperm morphology of infertile patients. J Assist Reprod Genet.

[B32] Mangoli E, Khalili MA, Talebi AR, Kalantar SM, Montazeri F, Agharahimi A, et al (2020). Association between early embryo morphokinetics plus transcript levels of sperm apoptotic genes and clinical outcomes in IMSI and ICSI cycles of male factor patients. J Assist Reprod Genet.

